# Natural Chromosome-Chromid Fusion across rRNA Operons in a *Burkholderiaceae* Bacterium

**DOI:** 10.1128/spectrum.02225-21

**Published:** 2022-01-05

**Authors:** Jiro F. Mori, Robert A. Kanaly

**Affiliations:** a Graduate School of Nanobiosicences, Yokohama City University, Yokohama, Japan; University of Minnesota

**Keywords:** chromid, chromosomal fusion, *Cupriavidus*, genomic rearrangement

## Abstract

Chromids (secondary chromosomes) in bacterial genomes that are present in addition to the main chromosome appear to be evolutionarily conserved in some specific bacterial groups. In rare cases among these groups, a small number of strains from *Rhizobiales* and *Vibrionales* were shown to possess a naturally fused single chromosome that was reported to have been generated through intragenomic homologous recombination between repeated sequences on the chromosome and chromid. Similar examples have never been reported in the family *Burkholderiaceae*, a well-documented group that conserves chromids. Here, an in-depth genomic characterization was performed on a *Burkholderiaceae* bacterium that was isolated from a soil bacterial consortium maintained on diesel fuel and mutagenic benzo[*a*]pyrene. This organism, Cupriavidus necator strain KK10, was revealed to carry a single chromosome with unexpectedly large size (>6.6 Mb), and results of comparative genomics with the genome of C. necator N-1^T^ indicated that the single chromosome of KK10 was generated through fusion of the prototypical chromosome and chromid at the rRNA operons. This fusion hypothetically occurred through homologous recombination with a crossover between repeated rRNA operons on the chromosome and chromid. Some metabolic functions that were likely expressed from genes on the prototypical chromid region were indicated to be retained. If this phenomenon—the bacterial chromosome-chromid fusion across the rRNA operons through homologous recombination—occurs universally in prokaryotes, the multiple rRNA operons in bacterial genomes may not only contribute to the robustness of ribosome function, but also provide more opportunities for genomic rearrangements through frequent recombination.

**IMPORTANCE** A bacterial chromosome that was naturally fused with the secondary chromosome, or “chromid,” and presented as an unexpectedly large single replicon was discovered in the genome of Cupriavidus necator strain KK10, a biotechnologically useful member of the family *Burkholderiaceae*. Although *Burkholderiaceae* is a well-documented group that conserves chromids in their genomes, this chromosomal fusion event has not been previously reported for this family. This fusion has hypothetically occurred through intragenomic homologous recombination between repeated rRNA operons and, if so, provides novel insight into the potential of multiple rRNA operons in bacterial genomes to lead to chromosome-chromid fusion. The harsh conditions under which strain KK10 was maintained—a genotoxic hydrocarbon-enriched milieu—may have provided this genotype with a niche in which to survive.

## INTRODUCTION

Bacterial genomes that contain a secondary large replicon in addition to the main chromosome, so-called secondary chromosomes or chromids, appear to account for about 10% of all bacterial genomes ever sequenced, and therefore, they have been referred to as “uncommon but not rare” (1–4). Distinct from the smaller replicons, i.e., plasmids that are typically <350 kbp in size and carry only nonessential genes, chromids with at least 350 kbp in size carry essential genes and thus hypothetically have been evolutionarily conserved in genomes of some specific bacterial taxa. Also, contrasted with the main chromosome, chromids are known to be genetically malleable because they undergo higher rates of evolution due to more frequent gene acquisition (and possibly gene loss) through horizontal gene transfer (2, 5). Because chromids were presumably evolved from plasmids, rather than by excision from the main chromosome, through acquiring and conserving foreign genes and expressing functional genes differentially from the main chromosome in response to surrounding conditions, it is therefore likely that they contribute to cellular abilities to adapt to and inhabit new environments (2, 6).

Since the bacterial chromid was first discovered in Rhodobacter sphaeroides (7), it has mostly been found in the proteobacteria, especially *Rhizobiales* from alphaproteobacteria, *Burkholderiales* from betaproteobacteria, and *Vibrionales* from gammaproteobacteria (3, 4). Among them, examples from the family *Burkholderiaceae* were well documented because the genera *Burkholderia*, *Paraburkholderia*, *Cupriavidus*, and *Ralstonia* from this family appeared to conserve chromids in their genomes with almost no exceptions. The ecological significance of chromid conservation has also been discussed (reference 2 and references therein). Chromids from these genera carried functional gene sets that were likely not absolutely essential but, rather, benefited cells by allowing them to adapt to new environments, such as in the cases of genes responsible for cell motility (flagellar biosynthesis and chemotaxis) or genes involved in central carbon metabolism (the Entner-Doudoroff glycolytic pathway). From *Rhizobiales*, strains of a plant symbiont, Sinorhizobium meliloti, were reported to possess both a chromid and (mega)plasmids, and the specific evolutionary roles for these replicons were clarified; while the chromid had acquired and conserved genes related to environmental adaptation, the plasmids mostly contributed to structural fluidity for the emergence of new function and had acquired the symbiosis-related genes (8).

Another well-studied bacterial group that contains chromids is Vibrio cholerae from the *Vibrionales*, the etiological agent of cholera. This organism has been used as a model system to study bacterial chromosomal replication and maintenance and segregation of multipartite genomes (references 6, 9, and references therein). In previous studies a synthetic single chromosome of V. cholerae was constructed in which the main chromosome and chromid were artificially fused to investigate bacterial chromosomal replication and maintenance systems (10, 11). More intriguingly, in recent studies two strains of V. cholerae were discovered, of which the main chromosome and chromid of each strain had naturally fused and presented as a single larger chromosome (12, 13). These chromosomal fusions appeared to have occurred through homologous recombination between repeated insertion sequence (IS) elements or site-specific recombination between *dif* sites in the replication terminus region (9, 11). In fact, a similar example was reported from a strain of S. meliloti as well in the past, of which a fused single chromosome was also reported to have been generated through intragenomic homologous recombination between the repeated sequences (14). These research achievements implied that such chromosomal fusion events mediated by intragenomic homologous recombination may occur frequently in nature; however, to the best of our knowledge, whether this phenomenon has occurred in members of the *Burkholderiales* has not yet been studied.

Here, an in-depth genomic characterization of a new isolate from the family *Burkholderiaceae*, Cupriavidus necator strain KK10, was conducted. Members of the genus *Cupriavidus* conserve a chromid in their genomes, the sizes of which were comparable to the main chromosomes (2, 15–19). Strain KK10 was isolated from a diesel fuel-grown soil bacterial consortium, and its abilities for biotransforming single-ring aromatic hydrocarbons, including azaarenes (20–22), as well as production of polyhydroxyalkanoates (PHA), have been studied. Thus, the complete genome of this organism was recently sequenced (23) with the aim of expanding our understanding of this unique and potentially useful organism. As result, a single chromosome with an unexpectedly large size was discovered in strain KK10 (23), and the results of analyses conducted here may provide new insights into the occurrence of natural genomic rearrangements and evolution in the family *Burkholderiaceae*.

## RESULTS

### A massive chromosome in strain KK10.

**FIG 1:**
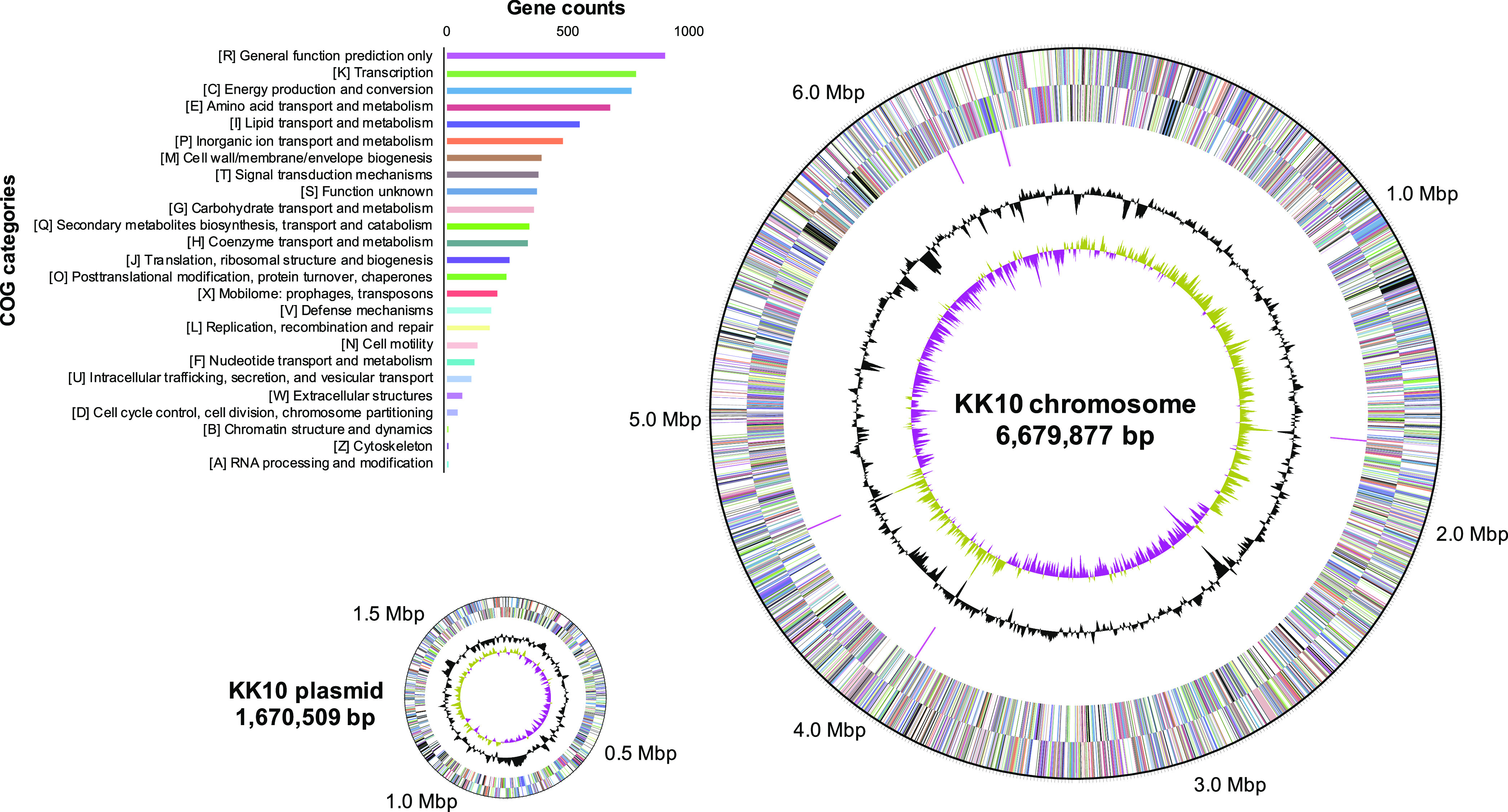
Circular maps of the Cupriavidus necator strain KK10 chromosome and plasmid with functional gene annotation. Rings from outside to the center are genes on forward strand and reverse strand (colored according to COG annotation categories, as listed in the bar graph with gene counts), rRNA operons (pink), GC content (gray), and GC skew (yellow and purple).

The hybrid assembly technique using the DNBSEQ short-read and GridION long-read sequencing data successfully provided a high-quality, circularized genome of strain KK10 (see Table S1 and Fig. S1 in the supplemental material). The genome of strain KK10, with a total size of 8,350,386 bp, consisted of two replicons—a chromosome (6,679,877 bp) and a (mega)plasmid (1,670,509 bp) (Fig. 1, Table 1)—that carried a total of 7,324 coding genes according to the Procaryotic Genome Annotation Pipeline (PGAP) annotation. Among the complete genomes of *Cupriavidus* strains available in databases, most strains had a chromid (<4 Mbp) in addition to the main chromosome (<5 Mbp), while only strain KK10 and another strain (Cupriavidus metallidurans strain Ni-2) (24) had a single chromosome of which the size was relatively large (>6 Mbp) while lacking a chromid (Table 1). The size of the KK10 chromosome is comparable in length to the largest single chromosomes within those known in *Burkholderiaceae* in the NCBI database (Burkholderia cenocepacia VC7848, 7.50 Mbp, and B. cenocepacia 895, 7.46 Mbp, which were isolated from clinical patients in unpublished studies). The KK10 chromosome possessed 5,957 coding genes and five rRNA gene (16S-5S-23S) operons (Fig. 1). Additionally, the plasmid of KK10, which possessed 1,367 coding genes, is 1.67 Mbp long; the sizes of the known plasmids of other *Cupriavidus* strains range from 30 kbp to 1.5 Mbp (Table 1).

**TABLE 1:** Summary of the chromosome and plasmid size of *Cupriavidus* genomes in the NCBI database

*Cupriavidus* strain	NCBI accession no	Chromosome size (bp)			Plasmid size (bp)				Total genome size (bp)
		1	2 (chromid)	Total	1	2	3	Total	
C. necator KK10	ASM1822372	6,679,877		6,679,877	1,670,509			1,670,509	8,350,386
C. necator N-1^T^	ASM21921	3,872,936	2,684,606	6,557,542	1,499,175	424,140		1,923,315	8,480,857
C. necator H16	ASM479872	4,049,965	2,912,457	6,692,422	452,139			452,139	7,414,561
C. necator NH9	ASM201192	4,347,557	3,395,604	7,743,161	426,602	77,172		503,774	8,246,935
C. nantogensis X1^T^	ASM159805	4,619,440	2,456,269	7,075,709					7,075,709
C. taiwanensis R1^T^	ASM6978	3,416,911	2,502,411	5,919,322	557,200				6,476,522
C. oxalaticus Ox1^T^	ASM1689438	3,885,446	2,809,304	6,694,750					6,694,750
C. malaysiensis USMAA1020^T^	ASM185432	4,383,984	3,388,586	7,772,570	176,879			176,879	7,949,449
C. gilardii CR3	ASM128146	3,539,530	2,039,213	5,578,743					5,578,743
C. pinatubonensis JMP134	ASM20387	3,806,533	2,726,152	6,532,685	634,917	87,688		722,605	7,255,290
C. basilensis 4G11	ASM83230	4,522,716	3,898,767	8,421,483					8,421,483
*C. metallidurans* CH34^T^	ASM19601	3,928,089	2,580,084	6,508,173	233,720	171,459		405,179	6,913,352
*C. metallidurans* Ni-2	ASM294476	6,543,877		6,543,877	197,700	180,778	30,753	409,231	6,953,108

### Comparative genomics between strains KK10 and N-1^T^.

**FIG 2:**
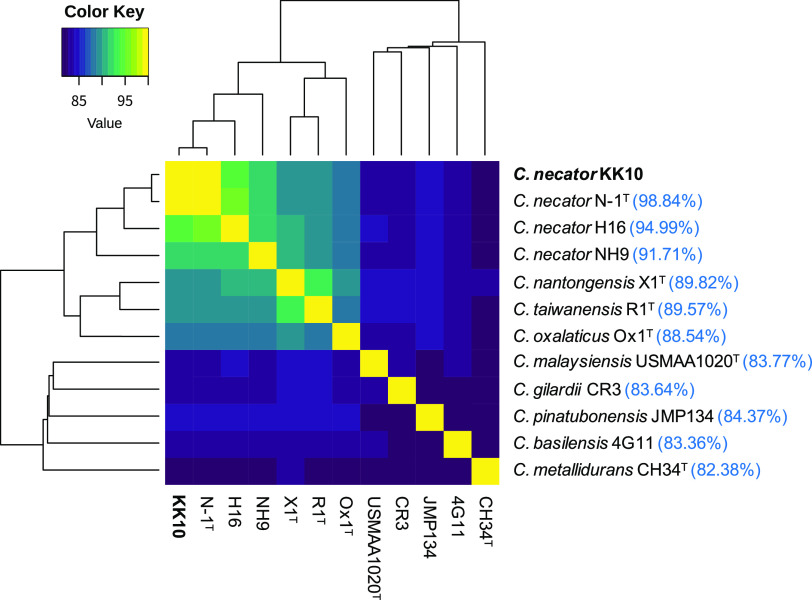
Heatmap of average nucleic acid identity (ANI) among C. necator strain KK10 and reference *Cupriavidus* genomes with cluster dendrograms. ANI values of each strain to strain KK10 are shown in parentheses in blue.

**FIG 3:**
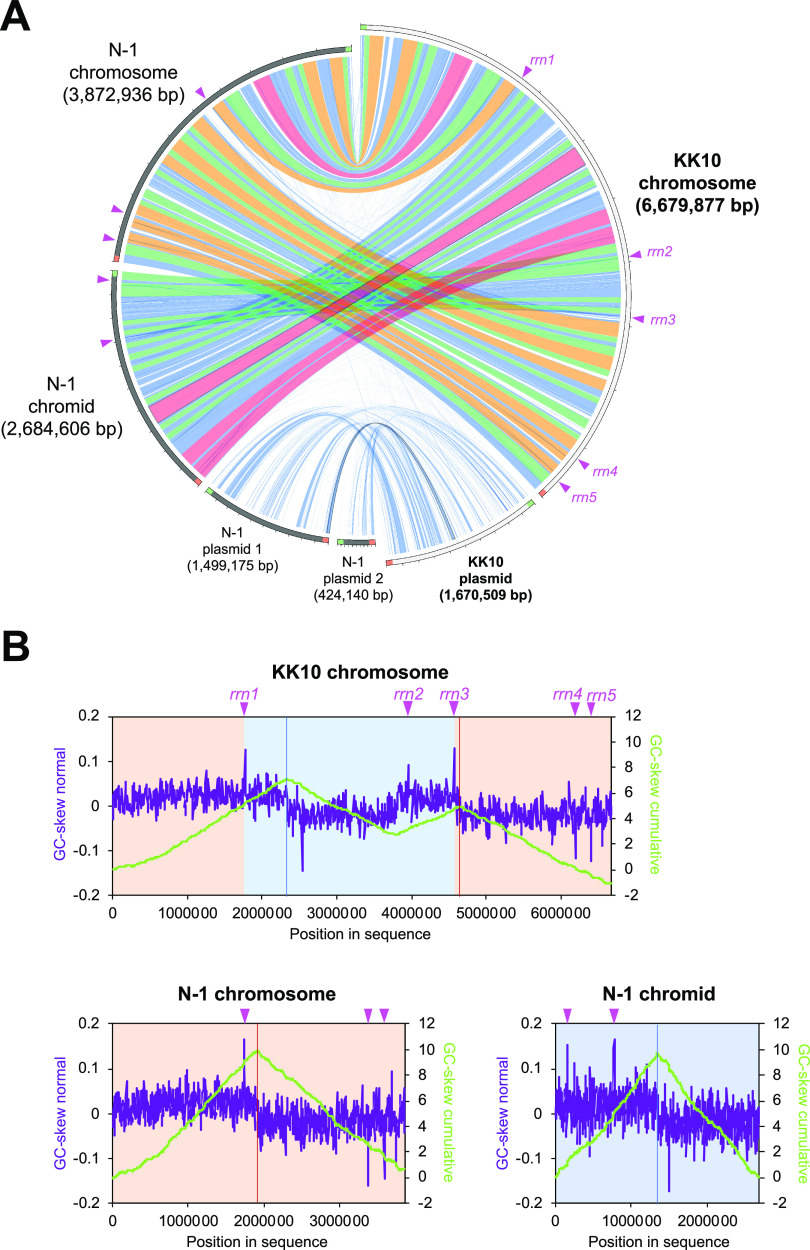
Sequencing comparisons between genomes of strain KK10 and N-1^T^. Pink arrowheads indicate the locations of rRNA operons (*rrn1* to -*5*). (A) Visualized sequencing similarities between KK10 and N-1 replicons. Local alignments are presented as ribbons with colors corresponding to the alignment bitscore in four quartiles (red, top 25%; orange, second 25%; green, third 25%; blue, worst 25%). (B) GC skew profiles of the chromosome of KK10 (above) and the chromosome and chromid of N-1 (below). GC skew peaks as potential replication termini are highlighted with red or blue lines.

Among the complete genomes of *Cupriavidus* strains available in databases, C. necator N-1^T^ showed the highest average nucleic acid identity (ANI; 98.84%; Fig. 2), followed by C. necator H16 (94.99%) and C. necator NH9 (91.71%). Within 5,957 coding genes in the KK10 chromosome, 5,480 genes (92.0%) were assigned as the orthologous genes shared with N-1, while the plasmid carried fewer orthologous genes (442 of 1,367 coding genes, 32.3%). The graphical genome comparisons between KK10 and N-1 shown in Fig. 3A clearly indicate synteny between their chromosomes. A genome region that showed synteny with the chromid of N-1 was distributed in the middle of the KK10 chromosome (position 1.77 Mb to 4.57 Mb; Fig. 3A) and suggested that the massive chromosome of KK10 was created through fusion of the prototypical chromosome and chromid; the reference genome of N-1 appeared to conserve the structures of prototypical replicons in the ancestral genome of KK10. The GC skew profile of the KK10 chromosome (Fig. 1 and 3B) clearly indicated two peaks originated from the prototypical chromosome and chromid. Interestingly, among five rRNA operons in the KK10 genome (*rrn1* to -*5*, shown as pink arrowheads in Fig. 3A), *rrn1* and *rrn3* were located exactly at both of the considered conjunction sites of the chromosome and chromid, providing evidence that the repetitive sequences of the rRNA operons induced fusion of the prototypical chromosome and chromid and resulted in the creation of a massive chromosome in KK10.

### Evidence of chromosome-chromid fusion in KK10 across the rRNA operons.

**FIG 4:**
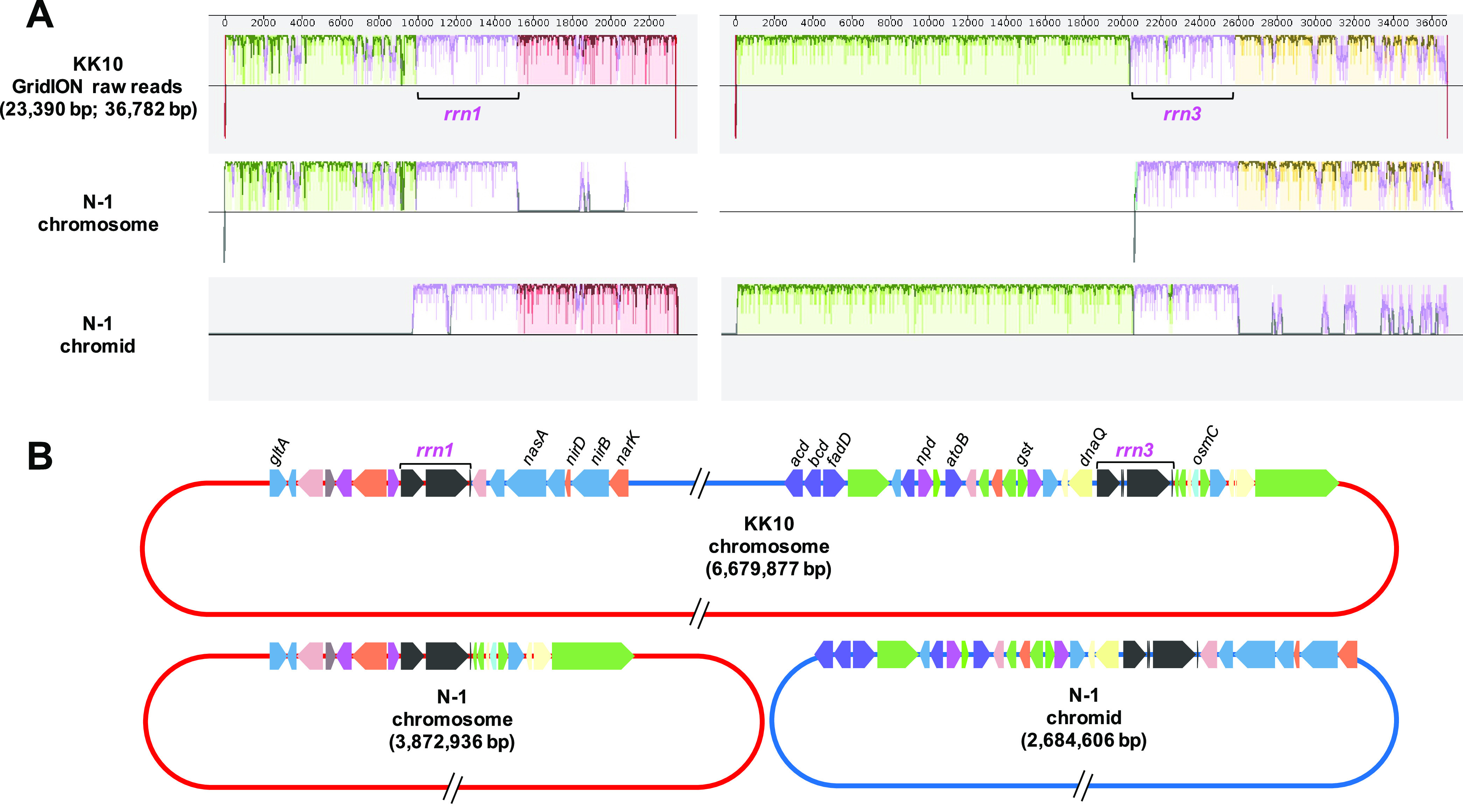
Sequencing alignments between the GridION long-read sequencing raw reads from the KK10 chromosome and the chromosome and chromid of N-1. (A) Two representative raw reads of the GridION long-read sequencing of the KK10 genome covering the upstream and downstream regions of the rRNA operons (*rrn1* or *rrn3*) provided evidence that chromosome-chromid fusion occurred across these rRNA operons. (B) Simplified gene map indicating the upstream and downstream regions of the rRNA operons; *gltA*, citrate synthase; *nasA*, assimilatory nitrate reductase catalytic subunit; *nirB*/*nirD*, assimilatory nitrite reductase large/small subunits; *narK*, nitrate/nitrite transporter; *acd*, acyl-CoA dehydrogenase; *bcd*, butyryl-CoA dehydrogenase; *fadD*, long-chain acyl-CoA synthetase; *npd*, nitronate monooxygenase; *atoB*, acetyl-CoA acetyltransferase; *gst*, glutathione *S*-transferase; *dnaQ*, DNA polymerase-3 subunit epsilon; *osmC*, organic hydroperoxide reductase. Functional genes are colored according to COG annotation categories.

The presence of repetitive multiple rRNA operons conserved in bacterial genomes often causes the fragmentation and/or misassembly of the genome sequencing reads, especially in cases where only a short-read sequencing method was applied and repeat sequences such as rRNA operons were longer than the read lengths (25, 26). To eliminate possibilities of misassemblies across rRNA operons and provide evidence that chromosome-chromid fusion occurred in the KK10 genome, raw data obtained from the GridION long-read sequencing was analyzed in detail. Multiple raw reads with lengths of 9,000 to 55,000 bp which provided coverage across the rRNA operons *rrn1* and *rrn3* (approximately 5,000 bp) located at the potential conjunction points were found (Fig. S2). Sequencing comparisons between these raw reads and the chromosome and chromid of N-1 clearly indicated that the regions upstream and downstream from *rrn1* and *rrn3* were swapped (Fig. 4A) without any exceptions (Fig. S2). Functional genes located upstream and downstream from *rrn1* and *rrn3* were perfectly conserved between KK10 and N-1 and involved genes potentially responsible for DNA replication/repair (DNA polymerase-3 subunit epsilon), nitrogen assimilation (nitrate/nitrite reductase/transporter, nitronate monooxygenase), fatty acid degradation (acyl-CoA synthetase/dehydrogenase, acetyl-CoA acetyltransferase), oxidative stress response (organic hydroperoxide reductase, glutathione *S*-transferase), and citrate synthesis (Fig. 4B).

### Characterizations of functional genes located on the KK10 chromosome.

**FIG 5:**
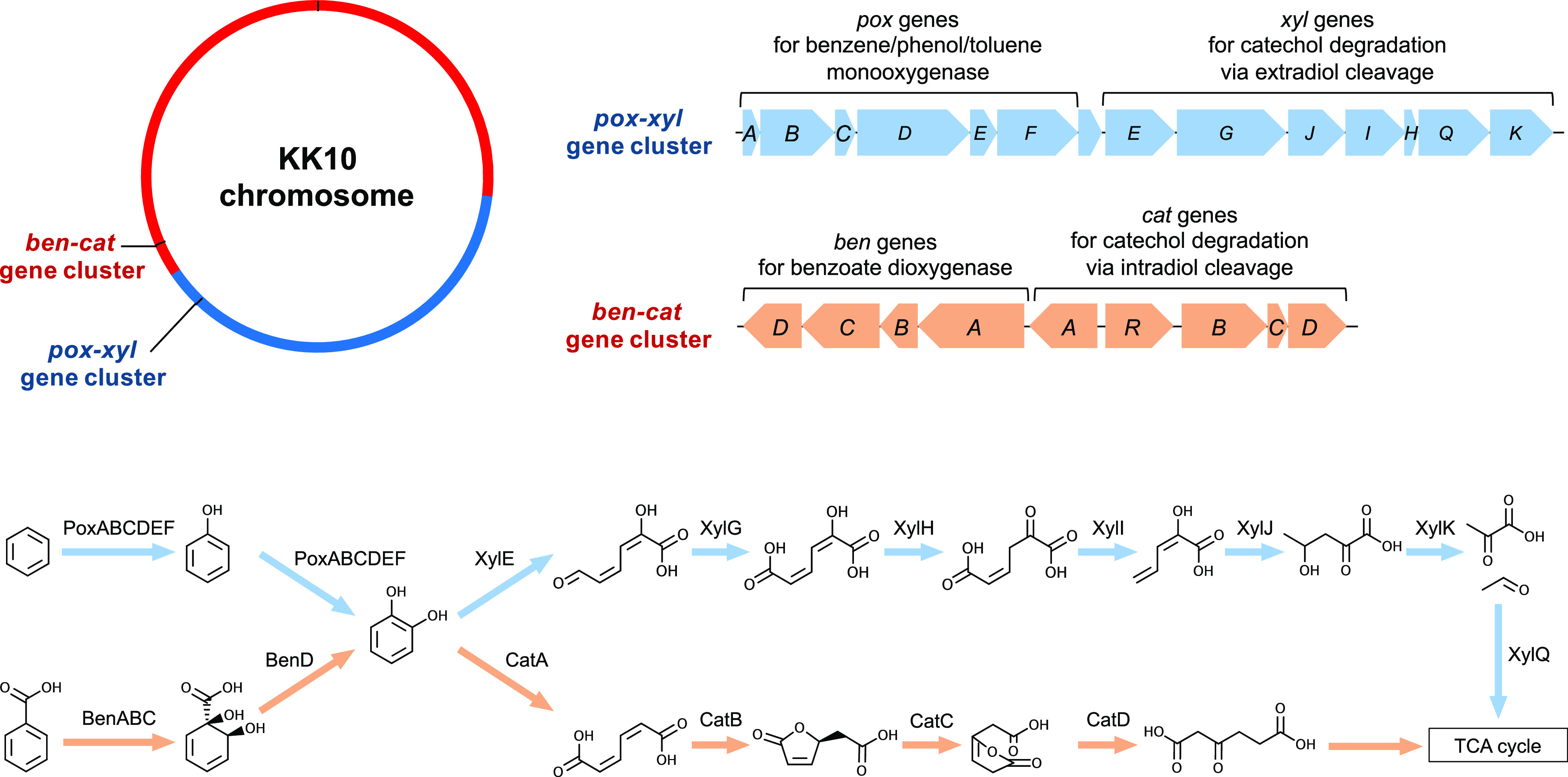
Gene clusters responsible for biotransformation of aromatic hydrocarbons conserved in the KK10 chromosome. The *pox*-*xyl* gene cluster encoding proteins for benzene/phenol/toluene degradation was located in a region that originated from the prototypical chromid (blue), while the *ben*-*cat* gene cluster for benzoic acid degradation was located in a region on the prototypical main chromosome (red).

Two gene clusters that were widely conserved in *Cupriavidus* strains and potentially responsible for aromatic hydrocarbon degradation were found in the KK10 chromosome (Fig. 5; Table S2). One of these gene clusters that contained *poxABCDEF* genes encoding benzene/phenol/toluene monooxygenase and *xylEGHIJKQ* genes for catechol degradation via an extradiol ring-cleavage pathway was characterized to be responsible for cell growth on benzene/phenol/toluene degradation (27) and was previously confirmed to enable *Cupriavidus* strains to grow on these hydrocarbon substrates as the sole carbon and energy sources (28). Another gene cluster, which consisted of *benABCD* genes encoding benzoate dioxygenase and *catABCD* genes for catechol degradation via an intradiol ring-cleavage pathway, was considered to be responsible for growth on benzoic acid (29). The *pox*-*xyl* gene cluster was located in a region of the KK10 chromosome that originated from the prototypical chromid (position 4.32 Mb to 4.33 Mb), and the *ben*-*cat* gene cluster was found in the region from the prototypical main chromosome (position 4.73 Mb to 4.74 Mb; Fig. 5, Table S2). Consistent with the findings of these functional genes in the KK10 genome, KK10 cells were confirmed to grow on benzene and benzoic acid as the sole carbon and energy sources through growth assays (Fig. S3).

## DISCUSSION

### Hypothetical mechanisms for the chromosome-chromid fusion across the rRNA operons.

**FIG 6:**
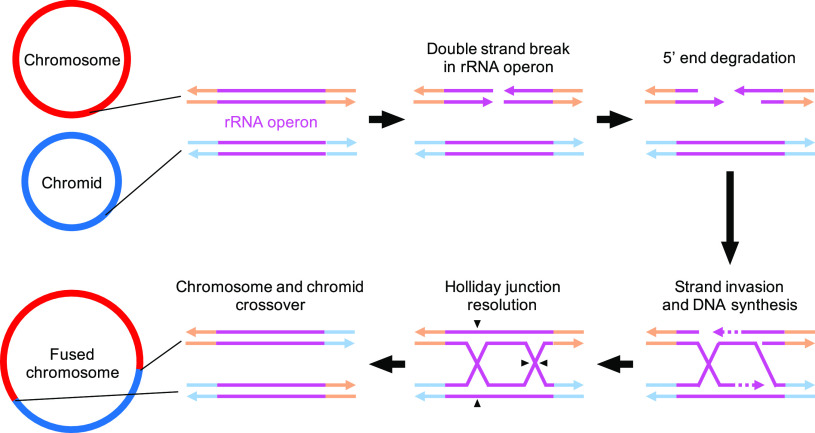
Hypothetical schematic model for the mechanism of chromosome-chromid fusion across rRNA operons in the KK10 genome. The process was initiated by a double-strand break in an rRNA operon (in either the prototypical chromosome or chromid). Through the typical repairing process of the double-strand break (generation of single-strand DNA [ssDNA] by 5′-end degradation, strand invasion, and DNA synthesis) and according to if the Holliday junctions were resolved via cleavage in the opposite orientations (black arrowheads), the regions upstream and downstream from the rRNA operon were swapped, resulting in a fused chromosome across the rRNA operons.

*Cupriavidus* genomes in the databases appeared to possess 4 to 7 rRNA operons that were located in both chromosomes and chromids. These repeated operons provide more opportunities for intragenomic recombination resulting in rearrangement of bacterial genomes (30), and indeed, repeated rRNA operons have been known to mediate chromosomal rearrangements in Salmonella (31–34). Previous studies proposed that the repeated sequences of multiple rRNA operons were conserved through concerted evolution among these operons, thereby avoiding divergence caused by frequent recombination, eliminating mutations and repairing possible double-strand breaks (35). Therefore, the chromosome-chromid fusion in the KK10 genome may be described by an intragenomic (i.e., between the main chromosome and chromid) homologous recombination mechanism with a typical crossover event through double-strand break repair in an rRNA operon via resolution of a double Holliday junction in opposite orientations (Fig. 6). Functional gene homologs encoding enzymes known to be responsible for DNA double-strand break repair, such as the helicase-nuclease complex AddAB (36), recombination protein RecA, Holliday junction helicase, and resolvase RuvABC and RecG, were all conserved in the KK10 chromosome. Another potential mechanism by which to explain the observed phenomenon is through template switching during replication resulting in a single circular chromosome (37). Notably, *rrn1* in the KK10 chromosome, which served as the conjunction point, was located close to the considered replication terminus of the prototypical chromosome, where there is a higher likelihood of mutations, chromosomal fusion, and overall spontaneous fragility compared to regions nearer to the replication origin (11, 38, 39). From this perspective, it is worth considering that strain KK10 had been maintained as a member of a bacterial consortium for many years on a diesel fuel carbon source and the mutagenic polycyclic aromatic hydrocarbon (PAH) benzo[*a*]pyrene (40). These harsh conditions may have contributed to increasing the possibilities of DNA damage and mutations in the members of the community (41, 42). It is still unclear if replication from the origin sites of the prototypical chromid is still functional or suppressed (6) in the fused single chromosome, and further investigations, such as marker frequency analysis (43), shall be required for clarification.

### Potential effects of the chromosome-chromid fusion on the gene functions.

Previous studies observed that the functional genes on the chromid appeared to be expressed niche-specifically and differentially from the genes on the main chromosome (6); therefore, genome rearrangements through the chromosomal (chromosome-chromid) fusion event may influence, or potentially even silence, the expression of functional genes on the prototypical chromid. In contrast, a previous study of Burkholderia cenocepacia proposed that translocations of a gene segment from a small replicon to the main chromosome increased their expression (44). Thus, a remaining question is how the expression behavior of functional genes in the KK10 chromosome were affected because of the chromosome-chromid fusion.

The *pox*-*xyl* gene cluster that was located in the region that originated from the prototypical chromid of KK10 and the *ben*-*cat* gene cluster that was found in the region from the prototypical main chromosome may each serve as potential indicators for expression of functional genes derived from each of the chromosomal regions. KK10 cells were confirmed to grow on benzene and benzoic acid here (Fig. S3). In a previous study KK10 grew on benzoic acid and salicylic acid, and the downstream metabolite of these compounds, catechol, was detected (20). A gene considered to be responsible for salicylic acid utilization, which encodes salicylate 1-hydroxylase, which transforms salicylic acid to catechol (28), was also found in the prototypical chromid region (position 4.55 Mb). Thus, the functional gene sets that originated from either the prototypical chromid or the prototypical main chromosome both appeared to be expressed and functioning, at least at certain levels that were sufficient to grow under the conditions tested unless other entirely unknown enzymes were functioning. Another potential indicator for the expression of functional genes includes gene sets responsible for cell motility. Gene operons encoding proteins related to flagellar biosynthesis and chemotaxis were widely conserved among *Cupriavidus* strains, and these were all located on the chromid (2). In the KK10 genome, these conserved gene sets were also found in the region that originated from the prototypical chromid (Table S3), and together with other strains previously reported (45–47), cell motility of KK10 was confirmed by microscopic observation. These results suggested that for at least some of the functional genes that were conserved in the region that originated from the prototypical chromid of KK10, their expression was not silenced.

These targeted functional genes that originated from the prototypical chromid of KK10 appeared to retain their functions; however, expression of other functions such as nitrogen assimilation or fatty acid metabolism, of which responsible genes were located close the fusion sites (Fig. 4B), could be more affected through this genomic rearrangement. Therefore, further transcriptome-based analyses and comparisons with other *Cupriavidus* strains shall be required to evaluate in detail how the expression levels of these functional genes and the cell survival rates were influenced through the chromosome-chromid fusion event.

### Frequency and ecological relevance of the chromosome-chromid fusion event.

The GC skew profiles of chromosomes with a size of >6.0 Mbp from other *Burkholderiaceae* strains, Cupriavidus metallidurans Ni-2 and Burkholderia cepacia LO6 (both sequenced using the PacBio RS II system) (24, 48), indicated that their chromosomes had multiple GC skew peaks, similar to the KK10 chromosome, and these results suggested that they contained regions that originated from multiple prototypical replicons, i.e., the main chromosome, chromid, and possibly, plasmids (Fig. S4). These are in contrast to the chromosome from a member of the genus *Pandoraea* (P. norimbergensis DSM11628; Fig. S4), which is known to lack a chromid in its genome and conserve relatively larger single chromosomes than those of other genera that widely conserve a chromid (2). Based on currently available database information, it is not certain whether these chromosomal structures from *C. metallidurans* Ni-2 and B. cepacia LO6 resulted from a naturally occurring chromosome-chromid fusion or from sequencing misassemblies. Further genome comparison between strain Ni-2 and *C. metallidurans* strain CH34^T^ (as a reference) indicated that the locations of potential chromosome-chromid fusion did not match the positions of their rRNA operons (Fig. S5), indicating that another unknown mechanism may have mediated the chromosome-chromid fusion unless it was caused by sequencing misassemblies.

Naturally occurring chromosome-chromid fusion in bacterial genomes has rarely been found and thus was referred to as an “exceptional” case in the bacterial groups that conserve chromids in their genomes (6, 9). Therefore, even though the bacterial chromosomal fusion mediated by homologous recombination or replication template switching may occur frequently in nature, the variants generated through such fusion (and possible excision) events may rarely outcompete other wild-type cells in ecosystems (6). However, interestingly, these bacterial genotypes may have successfully occupied niches in unique, specialized environments; the V. cholerae strains and B. cepacia LO6 discussed above were isolated from clinical samples, and *C. metallidurans* Ni-2 and strain KK10 were isolated from laboratory-maintained bacterial consortia. Fusion of the chromid into the main chromosome may have limited the chances of foreign gene acquisition into the genome due to the lower rates of horizontal gene transfers into the main chromosome compared to those into chromids (2). In contrast, it may have provided benefit to cells by stabilizing and conserving efficient functional genes that were carried on the chromid. From this perspective, the relatively large size (1.67 Mbp) of the KK10 plasmid may have resulted from the higher necessity for acquiring foreign genes into the plasmid instead of the chromid.

In summary, this study reported a new occurrence of bacterial chromosomal fusion with another replicon that was potentially mediated by intragenomic homologous recombination between repeated rRNA operons. Naturally occurring chromosome-chromid fusion by the same mechanism has not been previously studied, to the best of our knowledge, and it is still uncertain how common this phenomenon may be among prokaryotes. Intriguingly, the inverse phenomenon was previously reported in Salmonella; homologous recombination between rRNA operons generated a new plasmid from the main chromosome (49). Nonetheless, if these processes are occurring more than has been understood, the presence of multiple rRNA operons conserved in bacterial genomes may not only contribute to the robustness of the function of the ribosome, but also provide more opportunities for genomic rearrangements and evolution through frequent homologous recombination. Additionally, the results of this investigation also emphasize that long-read sequencing analyses may be rigorously applied to fully resolve a complete bacterial genome to avoid misassemblies that may occur at rRNA operons. Although short-read sequencing has been widely applied for bacterial genome sequencing, this work serves as a cautionary tale that these short reads are insufficient to differentiate between fused and unfused genomic structures. Future studies of the fused chromosome of strain KK10 may provide further information about the ecological relevance and significance of the bacterial chromosomal fusion, including how the fusion event positively or negatively affects bacterial survival in ecosystems.

## MATERIALS AND METHODS

### Bacterial strain and culture conditions.

Cupriavidus necator strain KK10 was isolated from a diesel fuel-degrading bacterial consortium that originated from cattle pasture soil from the Gulf region of Texas by streaking on solid medium with a heavy fraction of diesel fuel as the carbon and energy source (21, 22, 40, 50, 51). Strain KK10 was routinely cultured on 20 mM glycerol in Stainier’s basal medium (SBM) at 30°C with rotary shaking at 150 rpm in the dark. The growth capability of strain KK10 on the aromatic hydrocarbon substrates as sole carbon and energy sources was tested by incubating bacterial cells under the same conditions with 50 mg L^−1^ of selected substrates in 20 mL of SBM in 100-mL-volume conical flasks that were stoppered with silicone plugs. Cell growth was evaluated by measuring the optical density of the cultures at 600 nm, and light microscopy was utilized to observe motility and cell multiplication using an Eclipse E800 system (Nikon, Tokyo, Japan).

### Complete genome sequencing of strain KK10.

The complete genome sequence of strain KK10 was previously announced with detailed information on the sequencing method (23). In brief, genomic DNA of strain KK10 extracted from the cells grown on 20 mM glycerol for 3 days was sequenced employing a hybrid assembly of short-read (DNBSEQ-G400; MGI Tech, Shenzhen, China) and long-read (GridION X5; Oxford Nanopore Technologies, Oxford, UK) sequencing technologies. For the GridION sequencing, a library was created using a ligation sequencing kit (SQK-LSK109; after adapter ligation, >3-kb fragments were enriched). The long-read sequences that were obtained using R9.4.1 flow cells were base-called using Guppy v. 4.0.11 and then trimmed and quality-filtered using Porechop v. 0.2.3 and Filtlong v. 0.2.0 (>1 kb). After trimming and quality filtering of the raw reads obtained from each sequencing platform, the complete genome sequence was determined through *de novo* assembly using Unicycler v. 0.4.7 (52) and was validated using Bandage v. 0.8.1 (Fig. S1) (53). GenSkew v. 1.0 was used to calculate the GC skew and to approximate the origin and terminus of replication.

### Functional gene profiling and comparative genomics.

Genome annotation was performed through the annotation pipelines provided by NCBI (PGAP v. 5.2) and JGI (Integrated Microbial Genomes [IMG] annotation pipeline v. 5.0.20). The reference *Cupriavidus* genome sequences deposited in the NCBI and IMG databases were used for comparative genomics. ANI between strain KK10 and reference *Cupriavidus* strains was determined using fastANI v. 1.32 (54), and a heatmap with cluster dendrograms was created with the heatmap function in R. Sequence similarity between the strain KK10 genome and reference genomes was visualized using Circoletto (http://tools.bat.infspire.org/circoletto/) (55), an online tool based on Circos (56), and Mauve v. 2.4.0 (57). SonicParanoid v. 1.3.5 (58) was used to identify the orthologous genes between the KK10 genome and reference genomes.

### Data availability.

The sequencing assembly and raw data for the KK10 genome are all available in public databases, under the accession numbers CP073677 and CP073678 in NCBI GenBank and under accession number 2913661577 in the IMG/MER. The raw SRA sequences are available under accession numbers SRR14308055 and SRR14308056, which are under BioProject number PRJNA722091 and BioSample number SAMN18744514.

## References

[1] diCenzo CG, Finan MT. 2017. The divided bacterial genome: structure, function, and evolution. Microbiol Mol Biol Rev 81:e00019-17. doi:10.1128/MMBR.00019-17. 28794225 PMC5584315

[2] diCenzo GC, Mengoni A, Perrin E. 2019. Chromids aid genome expansion and functional diversification in the family Burkholderiaceae. Mol Biol Evol 36:562–574. doi:10.1093/molbev/msy248. 30608550

[3] Mergeay M, Van Houdt R. 2021. Plasmids as secondary chromosomes, p 1–5. In Bell E (ed), Molecular life sciences: an encyclopedic reference. Springer, New York, NY.

[4] Harrison PW, Lower RPJ, Kim NKD, Young JPW. 2010. Introducing the bacterial ‘chromid’: not a chromosome, not a plasmid. Trends Microbiol 18:141–148. doi:10.1016/j.tim.2009.12.010. 20080407

[5] Cooper VS, Vohr SH, Wrocklage SC, Hatcher PJ. 2010. Why genes evolve faster on secondary chromosomes in bacteria. PLoS Comput Biol 6:e1000732. doi:10.1371/journal.pcbi.1000732. 20369015 PMC2848543

[6] Sozhamannan S, Waldminghaus T. 2020. Exception to the exception rule: synthetic and naturally occurring single chromosome Vibrio cholerae. Environ Microbiol 22:4123–4132. doi:10.1111/1462-2920.15002. 32237026

[7] Suwanto A, Kaplan S. 1989. Physical and genetic mapping of the Rhodobacter sphaeroides 2.4.1 genome: presence of two unique circular chromosomes. J Bacteriol 171:5850–5859. doi:10.1128/jb.171.11.5850-5859.1989. 2808300 PMC210445

[8] Galardini M, Pini F, Bazzicalupo M, Biondi EG, Mengoni A. 2013. Replicon-dependent bacterial genome evolution: the case of Sinorhizobium meliloti. Genome Biol Evol 5:542–558. doi:10.1093/gbe/evt027. 23431003 PMC3622305

[9] Xie G, Johnson SL, Davenport KW, Rajavel M, Waldminghaus T, Detter JC, Chain PS, Sozhamannan S. 2017. Exception to the rule: genomic characterization of naturally occurring unusual Vibrio cholerae strains with a single chromosome. Int J Genomics 2017:8724304. doi:10.1155/2017/8724304. 28951866 PMC5603330

[10] Val M-E, Skovgaard O, Ducos-Galand M, Bland MJ, Mazel D. 2012. Genome engineering in Vibrio cholerae: a feasible approach to address biological issues. PLoS Genet 8:e1002472. doi:10.1371/journal.pgen.1002472. 22253612 PMC3257285

[11] Val M-E, Kennedy SP, Soler-Bistué AJ, Barbe V, Bouchier C, Ducos-Galand M, Skovgaard O, Mazel D. 2014. Fuse or die: how to survive the loss of Dam in Vibrio cholerae. Mol Microbiol 91:665–678. doi:10.1111/mmi.12483. 24308271

[12] Chapman C, Henry M, Bishop-Lilly KA, Awosika J, Briska A, Ptashkin RN, Wagner T, Rajanna C, Tsang H, Johnson SL, Mokashi VP, Chain PSG, Sozhamannan S. 2015. Scanning the landscape of genome architecture of non-O1 and non-O139 Vibrio cholerae by whole genome mapping reveals extensive population genetic diversity. PLoS One 10:e0120311. doi:10.1371/journal.pone.0120311. 25794000 PMC4368569

[13] Johnson SL, Khiani A, Bishop-Lilly KA, Chapman C, Patel M, Verratti K, Teshima H, Munk AC, Bruce DC, Han CS, Xie G, Davenport KW, Chain P, Sozhamannan S. 2015. Complete genome assemblies for two single-chromosome Vibrio cholerae isolates, strains 1154-74 (serogroup O49) and 10432-62 (serogroup O27). Genome Announc 3:e00462-15. doi:10.1128/genomeA.00462-15. 25977434 PMC4432340

[14] Guo X, Flores M, Mavingui P, Fuentes SI, Hernández G, Dávila G, Palacios R. 2003. Natural genomic design in Sinorhizobium meliloti: novel genomic architectures. Genome Res 13:1810–1817. doi:10.1101/gr.1260903. 12902376 PMC403772

[15] Poehlein A, Kusian B, Friedrich B, Daniel R, Bowien B. 2011. Complete genome sequence of the type strain Cupriavidus necator N-1. J Bacteriol 193:5017. doi:10.1128/JB.05660-11. 21742890 PMC3165677

[16] Moriuchi R, Dohra H, Kanesaki Y, Ogawa N. 2019. Complete genome sequence of 3-chlorobenzoate-degrading bacterium Cupriavidus necator NH9 and reclassification of the strains of the genera Cupriavidus and Ralstonia based on phylogenetic and whole-genome sequence analyses. Front Microbiol 10:133. doi:10.3389/fmicb.2019.00133. 30809202 PMC6379261

[17] Mazhar SH, Herzberg M, Ben Fekih I, Zhang C, Bello SK, Li YP, Su J, Xu J, Feng R, Zhou S, Rensing C. 2020. Comparative insights into the complete genome sequence of highly metal resistant Cupriavidus metallidurans strain BS1 isolated from a gold-copper mine. Front Microbiol 11:47. doi:10.3389/fmicb.2020.00047. 32117100 PMC7019866

[18] Lykidis A, Pérez-Pantoja D, Ledger T, Mavromatis K, Anderson IJ, Ivanova NN, Hooper SD, Lapidus A, Lucas S, González B, Kyrpides NC. 2010. The complete multipartite genome sequence of Cupriavidus necator JMP134, a versatile pollutant degrader. PLoS One 5:e9729. doi:10.1371/journal.pone.0009729. 20339589 PMC2842291

[19] Ray J, Waters RJ, Skerker JM, Kuehl JV, Price MN, Huang J, Chakraborty R, Arkin AP, Deutschbauer A. 2021. Complete genome sequence of Cupriavidus basilensis 4G11, isolated from the Oak Ridge Field Research Center site. Genome Announc 3:e00322-15. doi:10.1128/genomeA.00322-15. PMC4432324 25977418

[20] Mori JF, Kanaly RA. 2020. Multispecies diesel fuel biodegradation and niche formation are ignited by pioneer hydrocarbon-utilizing proteobacteria in a soil bacterial consortium. Appl Environ Microbiol 87:e02268-20. doi:10.1128/AEM.02268-20. 33067200 PMC7755252

[21] Fukuoka K, Tanaka K, Ozeki Y, Kanaly RA. 2015. Biotransformation of indole by Cupriavidus sp. strain KK10 proceeds through N-heterocyclic- and carbocyclic-aromatic ring cleavage and production of indigoids. Int Biodeterior Biodegradation 97:13–24. doi:10.1016/j.ibiod.2014.11.007.

[22] Fukuoka K, Ozeki Y, Kanaly RA. 2015. Aerobic biotransformation of 3-methylindole to ring cleavage products by Cupriavidus sp. strain KK10. Biodegradation 26:359–373. doi:10.1007/s10532-015-9739-0. 26126873

[23] Mori JF, Nagai M, Kanaly RA. 2021. Complete genome sequence of Cupriavidus necator KK10, an azaarene-degrading and polyhydroxyalkanoate-producing soil bacterium. Microbiol Resour Announc 10:e00423-21. doi:10.1128/MRA.00423-21. 34264105 PMC8280871

[24] Lee S, Khanal A, Cho A-H, Lee H, Kang M-S, Unno T, Hur H-G, Lee J-H. 2019. Cupriavidus sp. strain Ni-2 resistant to high concentration of nickel and its genes responsible for the tolerance by genome comparison. Arch Microbiol 201:1323–1331. doi:10.1007/s00203-019-01700-5. 31297579

[25] Acuña-Amador L, Primot A, Cadieu E, Roulet A, Barloy-Hubler F. 2018. Genomic repeats, misassembly and reannotation: a case study with long-read resequencing of Porphyromonas gingivalis reference strains. BMC Genomics 19:54. doi:10.1186/s12864-017-4429-4. 29338683 PMC5771137

[26] Chen L-X, Anantharaman K, Shaiber A, Eren AM, Banfield JF. 2020. Accurate and complete genomes from metagenomes. Genome Res 30:315–333. doi:10.1101/gr.258640.119. 32188701 PMC7111523

[27] Kukor JJ, Olsen RH. 1991. Genetic organization and regulation of a meta cleavage pathway for catechols produced from catabolism of toluene, benzene, phenol, and cresols by Pseudomonas pickettii PKO1. J Bacteriol 173:4587–4594. doi:10.1128/jb.173.15.4587-4594.1991. 1856161 PMC208133

[28] Pérez-Pantoja D, De la Iglesia R, Pieper DH, González B. 2008. Metabolic reconstruction of aromatic compounds degradation from the genome of the amazing pollutant-degrading bacterium Cupriavidus necator JMP134. FEMS Microbiol Rev 32:736–794. doi:10.1111/j.1574-6976.2008.00122.x. 18691224

[29] Berezina N, Yada B, Lefebvre R. 2015. From organic pollutants to bioplastics: insights into the bioremediation of aromatic compounds by Cupriavidus necator. N Biotechnol 32:47–53. doi:10.1016/j.nbt.2014.09.003. 25252021

[30] Hashimoto JG, Stevenson BS, Schmidt TM. 2003. Rates and consequences of recombination between rRNA operons. J Bacteriol 185:966–972. doi:10.1128/JB.185.3.966-972.2003. 12533472 PMC142796

[31] Liu S-L, Sanderson KE. 1998. Homologous recombination between rrn operons rearranges the chromosome in host-specialized species of Salmonella. FEMS Microbiol Lett 164:275–281. doi:10.1111/j.1574-6968.1998.tb13098.x. 9682477

[32] Helm RA, Lee AG, Christman HD, Maloy S. 2003. Genomic rearrangements at rrn operons in Salmonella. Genetics 165:951–959. doi:10.1093/genetics/165.3.951. 14668356 PMC1462832

[33] Anderson P, Roth J. 1981. Spontaneous tandem genetic duplications in Salmonella typhimurium arise by unequal recombination between rRNA (rrn) cistrons. Proc Natl Acad Sci USA 78:3113–3117. doi:10.1073/pnas.78.5.3113. 6789329 PMC319510

[34] Liu SL, Sanderson KE. 1995. The chromosome of Salmonella paratyphi A is inverted by recombination between rrnH and rrnG. J Bacteriol 177:6585–6592. doi:10.1128/jb.177.22.6585-6592.1995. 7592437 PMC177512

[35] Espejo RT, Plaza N. 2018. Multiple ribosomal RNA operons in bacteria; their concerted evolution and potential consequences on the rate of evolution of their 16S rRNA. Front Microbiol 9:1232. doi:10.3389/fmicb.2018.01232. 29937760 PMC6002687

[36] Chédin F, Noirot P, Biaudet V, Ehrlich SD. 1998. A five-nucleotide sequence protects DNA from exonucleolytic degradation by AddAB, the RecBCD analogue of Bacillus subtilis. Mol Microbiol 29:1369–1377. doi:10.1046/j.1365-2958.1998.01018.x. 9781875

[37] Anand RP, Tsaponina O, Greenwell PW, Lee C-S, Du W, Petes TD, Haber JE. 2014. Chromosome rearrangements via template switching between diverged repeated sequences. Genes Dev 28:2394–2406. doi:10.1101/gad.250258.114. 25367035 PMC4215184

[38] Kivisaar M. 2019. Mutation and recombination rates vary across bacterial chromosome. Microorganisms 8:25. doi:10.3390/microorganisms8010025. 31877811 PMC7023495

[39] Mei Q, Fitzgerald DM, Liu J, Xia J, Pribis JP, Zhai Y, Nehring RB, Paiano J, Li H, Nussenzweig A, Hastings PJ, Rosenberg SM. 2021. Two mechanisms of chromosome fragility at replication-termination sites in bacteria. Sci Adv 7:eabe2846. doi:10.1126/sciadv.abe2846. 34144978 PMC8213236

[40] Kanaly RA, Bartha R, Watanabe K, Harayama S. 2000. Rapid mineralization of benzo[a]pyrene by a microbial consortium growing on diesel fuel. Appl Environ Microbiol 66:4205–4211. doi:10.1128/AEM.66.10.4205-4211.2000. 11010861 PMC92287

[41] Penning TM. 2014. Human aldo-keto reductases and the metabolic activation of polycyclic aromatic hydrocarbons. Chem Res Toxicol 27:1901–1917. doi:10.1021/tx500298n. 25279998 PMC4237494

[42] Takeshita T, Kanaly RA. 2019. In vitro DNA/RNA adductomics to confirm DNA damage caused by benzo[a]pyrene in the Hep G2 cell line. Front Chem 7:491. doi:10.3389/fchem.2019.00491. 31338364 PMC6629907

[43] Skovgaard O, Bak M, Løbner-Olesen A, Tommerup N. 2011. Genome-wide detection of chromosomal rearrangements, indels, and mutations in circular chromosomes by short read sequencing. Genome Res 21:1388–1393. doi:10.1101/gr.117416.110. 21555365 PMC3149504

[44] Morrow JD, Cooper VS. 2012. Evolutionary effects of translocations in bacterial genomes. Genome Biol Evol 4:1256–1262. doi:10.1093/gbe/evs099. 23160175 PMC3542574

[45] Makkar NS, Casida LE. 1987. Cupriavidus necator gen. nov., sp. nov.; a nonobligate bacterial predator of bacteria in soil. Int J Syst Evol Microbiol 37:323–326.

[46] Shamim S, Rehman A, Qazi MH. 2014. Swimming, swarming, twitching, and chemotactic responses of Cupriavidus metallidurans CH34 and Pseudomonas putida mt2 in the presence of cadmium. Arch Environ Contam Toxicol 66:407–414. doi:10.1007/s00244-013-9966-5. 24306627

[47] Wakimoto T, Nakagishi S, Matsukawa N, Tani S, Kai K. 2020. A unique combination of two different quorum sensing systems in the β-rhizobium Cupriavidus taiwanensis. J Nat Prod 83:1876–1884. doi:10.1021/acs.jnatprod.0c00054. 32484353

[48] Belcaid M, Kang Y, Tuanyok A, Hoang TT. 2015. Complete genome sequence of Burkholderia cepacia strain LO6. Genome Announc 3:e00587-15. doi:10.1128/genomeA.00587-15. 26067955 PMC4481279

[49] Blazey DL, Burns RO. 1983. recA-dependent recombination between rRNA operons generates type II F′ plasmids. J Bacteriol 156:1344–1348. doi:10.1128/jb.156.3.1344-1348.1983. 6196351 PMC217986

[50] Kanaly RA, Bartha R. 1999. Cometabolic mineralization of benzo[a]pyrene caused by hydrocarbon additions to soil. Environ Toxicol Chem 18:2186–2190. doi:10.1002/etc.5620181010. 29857609

[51] Kanaly RA, Watanabe K. 2004. Multiple mechanisms contribute to the biodegradation of benzo[a]pyrene by petroleum-derived multicomponent nonaqueous-phase liquids. Environ Toxicol Chem 23:850–856. doi:10.1897/03-191. 15095879

[52] Wick RR, Judd LM, Gorrie CL, Holt KE. 2017. Unicycler: resolving bacterial genome assemblies from short and long sequencing reads. PLoS Comput Biol 13:e1005595. doi:10.1371/journal.pcbi.1005595. 28594827 PMC5481147

[53] Wick RR, Schultz MB, Zobel J, Holt KE. 2015. Bandage: interactive visualization of de novo genome assemblies. Bioinformatics 31:3350–3352. doi:10.1093/bioinformatics/btv383. 26099265 PMC4595904

[54] Jain C, Rodriguez-R LM, Phillippy AM, Konstantinidis KT, Aluru S. 2018. High throughput ANI analysis of 90K prokaryotic genomes reveals clear species boundaries. Nat Commun 9:5114. doi:10.1038/s41467-018-07641-9. 30504855 PMC6269478

[55] Darzentas N. 2010. Circoletto: visualizing sequence similarity with Circos. Bioinformatics 26:2620–2621. doi:10.1093/bioinformatics/btq484. 20736339

[56] Krzywinski M, Schein J, Birol I, Connors J, Gascoyne R, Horsman D, Jones SJ, Marra MA. 2009. Circos: an information aesthetic for comparative genomics. Genome Res 19:1639–1645. doi:10.1101/gr.092759.109. 19541911 PMC2752132

[57] Darling AE, Mau B, Perna NT. 2010. progressiveMauve: multiple genome alignment with gene gain, loss and rearrangement. PLoS One 5:e11147. doi:10.1371/journal.pone.0011147. 20593022 PMC2892488

[58] Cosentino S, Iwasaki W. 2019. SonicParanoid: fast, accurate and easy orthology inference. Bioinformatics 35:149–151. doi:10.1093/bioinformatics/bty631. 30032301 PMC6298048

