# Supplementary material for: Natural Chromosome-Chromid Fusion across rRNA Operons in a Burkholderiaceae Bacterium
Source: Microbiol Spectr. 2022 Jan 5;10(1):e02225-21. doi: 10.1128/spectrum.02225-21 (PMC8729776; doi:10.1128/spectrum.02225-21)
Supplement: SUPPLEMENTAL FILE 1 — Supplemental material. Download SPECTRUM02225-21_Supp_1_seq10.pdf, PDF file, 4.9 MB [file spectrum02225-21_supp_1_seq10.pdf]

Supplementary Material

Microbiology Spectrum

Natural chromosome-chromid fusion across rRNA operons in a *Burkholderiaceae* bacterium

Jiro F. Mori and Robert A. Kanaly

Graduate School of Nanobiosicences, Yokohama City University, 22-2 Seto, Kanazawa-ku, Yokohama 236-0027, Japan

Corresponding author

Jiro F. Mori

Yokohama City University, Japan

morij@yokohama-cu.ac.jp

+81 45 787 2336

Table S1. Detailed information on the short-read and the long-read sequencing results.

Table S2. Presence of aromatic hydrocarbon degradation genes detected in the KK10 genome.

Table S3. Presence of flagellar biosynthesis and chemotaxis genes detected in the KK10 genome.

Figure S1. Visual validation of the circular chromosome and plasmid in the KK10 genome using Bandage.

Figure S2. Results of analyses of sequencing alignments between the assembled sequence of the KK10 chromosome and all GridION long-read sequencing raw reads that covered the upstream and downstream regions of the rRNA operons (*rrn1-5*).

Figure S3. Microscopic images of KK10 cells when grown on benzene or benzoic acid.

Figure S4. GC skew profiles of the chromosomes with the size of > 6.0 Mbp from the *Burkholderiaceae* strains.

Figure S5. Visualized sequencing similarities between the *C. metallidurans* Ni-2 and *C. metallidurans* $CH34^T$ genomes.

Table S1. Detailed information on the short-read and the long-read sequencing results.

**Number of reads**		**Average read length (bp)**		**Total read size (bp)**		**Assembly total size (bp)**	**Assembly GC content (%)**	**Assembly coverage**
DNBSEQ (short-read)	GridION (long-read)	DNBSEQ (short-read)	GridION (long-read)	DNBSEQ (short-read)	GridION (long-read)			
6,210,182	130,465	200	6,801	1,242,036,400	887,294,479	8,350,386	65.6	255

Table S2. Presence of aromatic hydrocarbon degradation genes detected in the KK10 genome.

Gene	EC	AA length	Locus	IMG Gene ID
Benzene/phenol/toluene monooxygenase *poxA*	1.14.13.244	74	4322149-4322373	2913665699
Benzene/phenol/toluene monooxygenase *poxB*	1.14.13.244	331	4322424-4323419	2913665700
Benzene/phenol/toluene monooxygenase *poxC*	1.14.13.244	93	4323452-4323733	2913665701
Benzene/phenol/toluene monooxygenase *poxD*	1.14.13.244	506	4323769-4325289	2913665702
Benzene/phenol/toluene monooxygenase *poxE*	1.14.13.244	119	4325321-4325680	2913665703
Benzene/phenol/toluene monooxygenase *poxF*	1.14.13.244	355	4325718-4326785	2913665704
Catechol 2,3-dioxygenase *xylE*	1.13.11.2	311	4327192-4328127	2913665706
2-hydroxymuconate-6-semialdehyde dehydrogenase *xylG*	1.2.1.85	484	4328243-4329697	2913665707
2-oxopent-4-enoate hydratase *xylJ*	4.2.1.80	260	4329707-4330489	2913665708
2-oxo-3-hexenedioate decarboxylase *xylI*	4.1.1.77	262	4330526-4331314	2913665709
4-oxalocrotonate tautomerase *xylH*	5.3.2.6	63	4331338-4331529	2913665710
Acetaldehyde dehydrogenase *xylQ*	1.2.1.10	314	4331555-4332499	2913665711
4-hydroxy-2-oxovalerate aldolase *xylK*	4.1.3.39	354	4332517-4333581	2913665712
Salicylate 1-monooxygenase *nahG*	1.14.13.1	378	4548682-4549818	2913665914
Dihydroxycyclohexadiene carboxylate dehydrogenase *benD*	1.3.1.25	261	4734552-4735337	2913666091
Benzoate 1,2-dioxygenase reductase subunit *benC*	1.14.12.10	339	4735355-4736374	2913666092
Benzoate 1,2-dioxygenase beta subunit *benB*	1.14.12.10	171	4736396-4736911	2913666093
Benzoate 1,2-dioxygenase alpha subunit *benA*	1.14.12.10	474	4736908-4738332	2913666094
Catechol 1,2-dioxygenase *catA*	1.13.11.	307	4738436-4739359	2913666095
Transcriptional regulator *catR*		305	4739507-4740424	2913666096
Muconate cycloisomerase *catB*	5.5.1.1	381	4740538-4741683	2913666097
Muconolactone D-isomerase *catC*	5.3.3.4	92	4741729-4742007	2913666098
3-oxoadipate enol-lactonase *catD*	3.1.1.24	260	4742021-4742803	2913666099

Table S3. Presence of flagellar biosynthesis and chemotaxis genes detected in the KK10 genome.

Gene	AA length	Locus	IMG Gene ID
Flagellin	435	3629025-3630332	2913665045
Flagellar protein *flaG*	125	3630470-3630847	2913665046
Flagellar hook-associated protein 2	468	3630881-3632287	2913665047
Flagellar protein *fliS*	141	3632326-3532751	2913665048
Flagellar protein *fliT*	113	3632753-3633094	2913665049
Flagellar biosynthesis protein	95	3634487-3634774	2913665051
Flagellar hook-basal body complex protein *fliE*	107	3634852-3635175	2913665052
Flagellar M-ring protein *fliF*	576	3635582-3637312	2913665053
Flagellar motor switch protein *fliG*	336	3637299-3638309	2913665054
Flagellar assembly protein *fliH*	270	3638302-3639114	2913665055
Flagellum-specific ATP synthase	491	3639107-3640582	2913665056
Flagellar protein *fliJ*	152	3640560-3641018	2913665057
Flagellar hook-length control protein *fliK*	474	3641045-3642469	2913665058
Methyl-accepting chemotaxis protein	568	4040767-4042473	2913665426
Two-component system chemotaxis response regulator *cheY*	130	4044710-4045102	2913665428
Purine-binding chemotaxis protein *cheW*	158	4045244-4045720	2913665429
Aerotaxis receptor	549	4045824-4047473	2913665430
Methyl-accepting chemotaxis protein	619	4047594-4049453	2913665431
Flagellar transcriptional activator *flhD*	105	4050718-4051035	2913665433
Flagellar transcriptional activator *flhC*	201	4051032-4051637	2913665434
Chemotaxis protein *motA*	287	4051757-4052620	2913665435
Chemotaxis protein *motB*	321	4052648-4053613	2913665436
Two-component system chemotaxis sensor kinase *cheA*	676	4053651-4055681	2913665437
Purine-binding chemotaxis protein *cheW*	164	4055724-4056218	2913665438
Chemotaxis protein methyltransferase *cheR*	303	4056249-4057160	2913665439
Chemotaxis protein *cheD*	216	4057160-4057810	2913665440
Two-component system chemotaxis response regulator *cheB*	360	4057834-4058916	2913665441
Two-component system chemotaxis response regulator *cheY*	128	4059005-4059391	2913665442
Chemotaxis protein *cheZ*	210	4059409-4060041	2913665443
Flagellar biosynthesis protein *flhB*	380	4062583-4063725	2913665447
Flagellar biosynthesis protein *flhA*	693	4063722-4065803	2913665448
Flagellar biosynthesis protein *flhF*	776	4065814-4068144	2913665449

(Continued on next page)

(Table S3, continued)

Gene	AA length	Locus	IMG Gene ID
Flagellar biosynthesis protein *flhG*	269	4068141-4068950	2913665450
RNA polymerase sigma factor for flagellar operon *fliA*	238	4069047-4069763	2913665451
Flagellar protein *flhE*	142	4069772-4070200	2913665452
Flagellar synthesis protein *flgN*	153	4070228-4070689	2913665453
Flagellar regulator or flagellin synthesis *flgM*	102	4070695-4071003	2913665454
Flagellar basal-body P-ring formation protein *flgA*	267	4071165-4071968	2913665455
Flagellar basal-body rod protein *flgB*	135	4072213-4072620	2913665456
Flagellar basal-body rod protein *flgC*	135	4072645-4073052	2913665457
Flagellar basal-body rod modification protein *flgD*	223	4073108-4073779	2913665458
Flagellar hook protein *flgE*	413	4073848-4075089	2913665459
Flagellar basal-body rod protein *flgF*	247	4075116-4075859	2913665460
Flagellar basal-body rod protein *flgG*	261	4075914-4076699	2913665461
Flagellar L-ring protein precursor *flgH*	232	4076711-4077409	2913665462
Flagellar P-ring protein precursor *flgI*	381	4077417-4078562	2913665463
Flagellar protein *flgJ*	347	4078565-4079608	2913665464
Flagellar hook-associated protein 1 *flgK*	641	4081130-4083055	2913665466
Flagellar hook-associated protein 3 *flgL*	409	4083102-4084331	2913665467
Flagellar biosynthesis protein *fliR*	261	4345129-4345914	2913665723
Flagellar biosynthesis protein *fliQ*	89	4345973-4346242	2913665724
Flagellar biosynthesis protein *fliP*	261	4346283-4347068	2913665725
Flagellar protein *fliO* / *fliZ*	151	4347065-4347520	2913665726
Flagellar motor switch protein *fliN* / *fliY*	149	4347517-4347966	2913665727
Flagellar motor switch protein *fliM*	339	4347959-4348978	2913665728
Flagellar protein *fliL*	163	4349038-4349529	2913665729

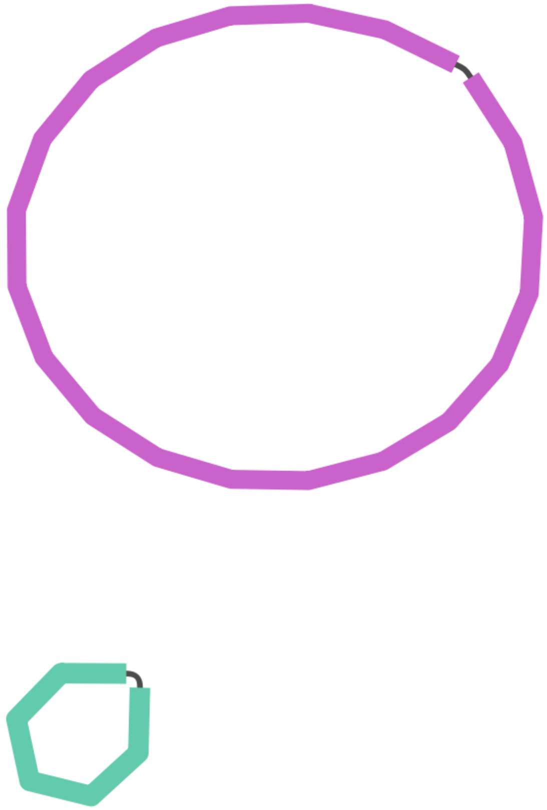

Figure S1. Visual validation of the circular chromosome and plasmid in the KK10 genome using Bandage.

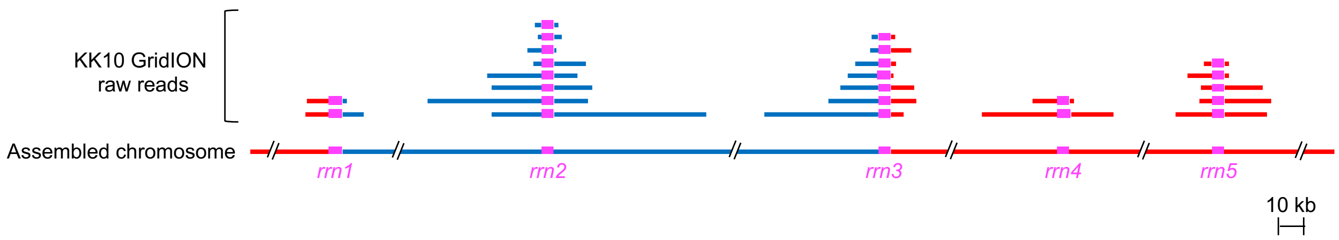

Figure S2. Results of analyses of sequencing alignments between the assembled sequence of the KK10 chromosome and all GridION long-read sequencing raw reads that covered the upstream and downstream regions of the rRNA operons (*rrn1*-*5*).

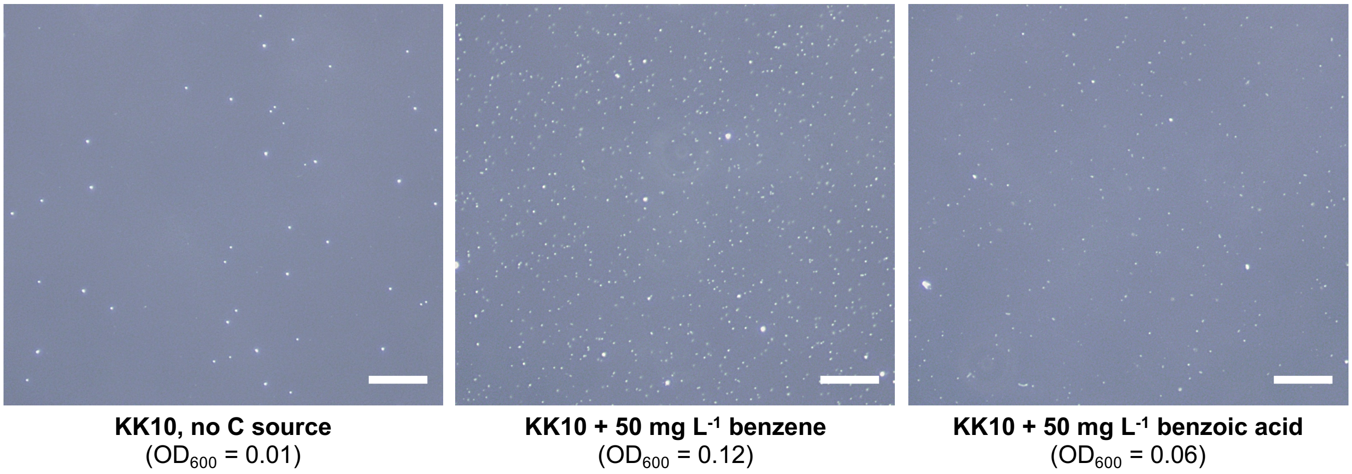

Figure S3. Microscopic images of KK10 cells when grown on benzene or benzoic acid. KK10 cell growth was confirmed after 4 days of incubation with 50 mg L^{-1} benzene (center) or 50 ppm benzoic acid (right) as sole carbon and energy sources. As a control, KK10 cells were incubated for 4 days without a carbon and energy source (left). Scale bars indicate 50 µm.

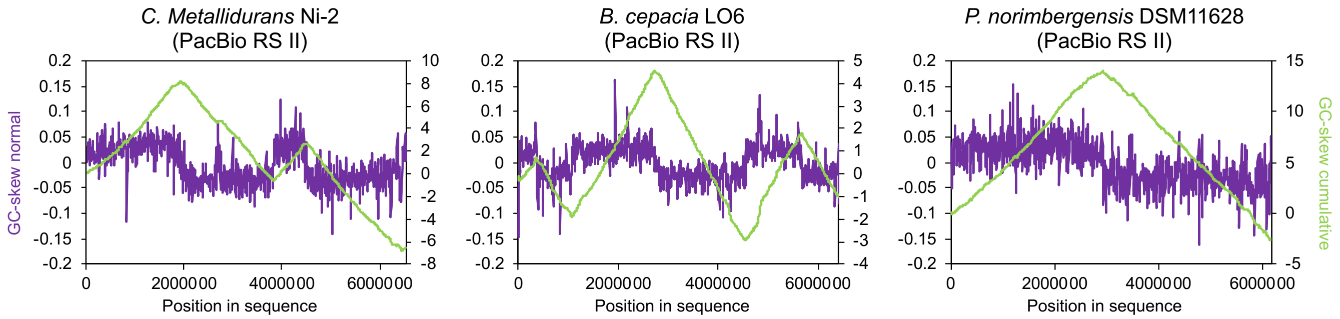

Figure S4. GC skew profiles of the chromosomes with the size of > 6.0 Mbp from the *Burkholderiaceae* strains.

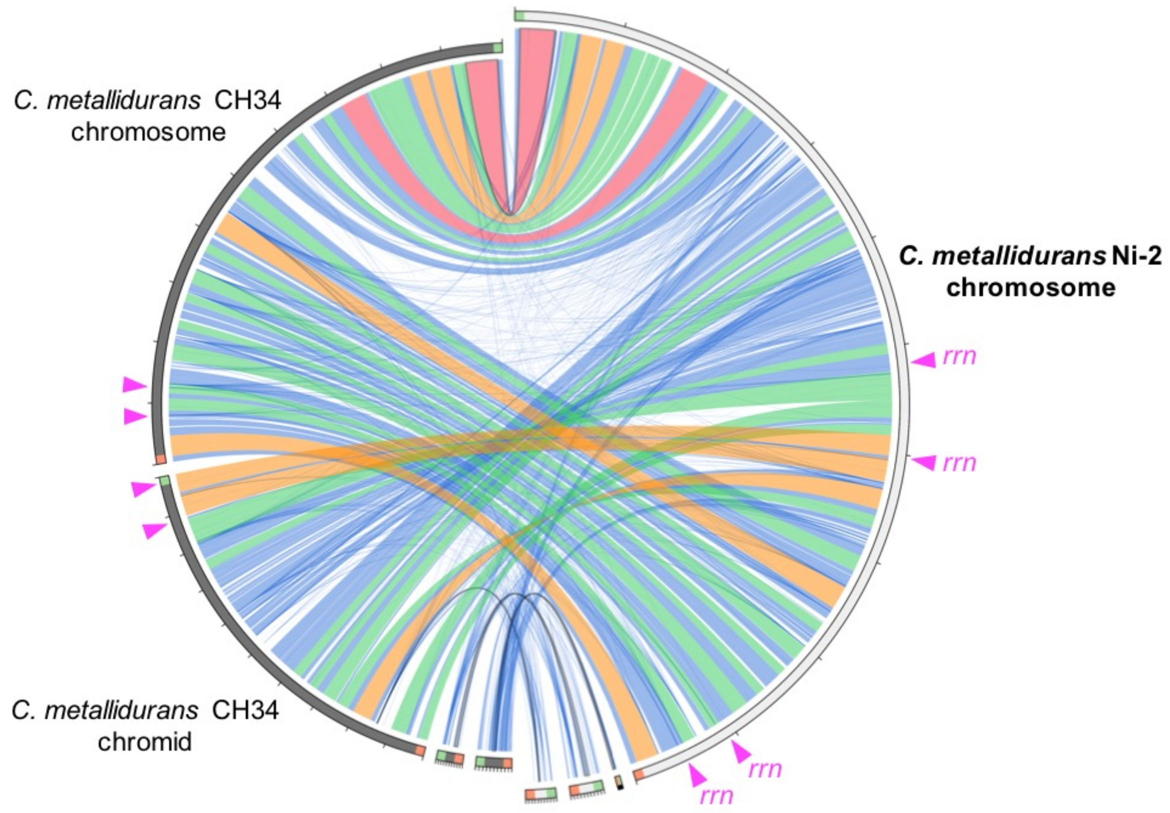

Figure S5. Visualized sequencing similarities between the *C. metallidurans* Ni-2 and *C. metallidurans* $CH34^T$ genomes.
